# Metallic Full-Length Ureteral Stents: Does Urinary Tract Infection Cause Obstruction?

**DOI:** 10.1100/tsw.2010.162

**Published:** 2010-08-17

**Authors:** James A. Brown, Christopher L. Powell, Kristopher R. Carlson

**Affiliations:** Department of Surgery, Division of Urology, Medical College of Georgia Hospitals and Clinics, Augusta, GA, USA

**Keywords:** ureteral stents, urinary tract infection, ureteral obstruction, ureteral compression

## Abstract

Metallic ureteral stents promise to offer superior upper urinary tract drainage with extended exchange intervals and freedom from extrinsic compression in patients with advanced malignancy or other significant obstructing retroperitoneal or pelvic processes. Existing literature indicates a variable experience with these relatively new devices, with some investigators reporting excellent results and long problem-free intervals, and others reporting less enthusiastic outcomes. We report a retrospective review of a series of five sequential patients undergoing placement of Resonance® (Cook Medical, Bloomington, IN) metallic ureteral stents for extrinsic ureteral compression refractory to placement of traditional (polymer) ureteral stents. Of five patients reviewed, three (60%) required additional operative intervention for stent migration or malposition. Four patients (80%) died of their primary malignancy <12 months after metallic stent placement. Four (80%) of five patients had obstruction of their stents demonstrated with nuclear renography and/or other imaging, and three (60%) required removal and alternative means of urinary tract drainage within 4 months of placement due to obstruction, intractable pain, or migration. Four patients (80%) had urinary tract infections (UTIs) within 4 months of stent placement. No obstruction was seen due to extrinsic ureteral compression after stent placement. Metallic ureteral stents may have utility for patients with pathological processes causing extrinsic ureteral compression refractory to the use of traditional polymer ureteral stents. However, metallic ureteral stents are not immune to obstruction, migration, and associated discomfort. Stent obstruction appears to be increased in patients with postoperative UTI.

## INTRODUCTION

As early as 1992, urologists and medical device manufacturers experimented with the use of metal alloys in stent construction to produce devices resistant to extrinsic ureteral compression[1]. Several studies demonstrated significant failure rates of polymer stents in the face of extrinsic malignant ureteral compression[2,3]. The Resonance® ureteral stent (Cook Medical, Bloomington, IN) was approved in 2007 for ureteral stenting in cases of extrinsic malignant compression. Limited data have been published to date on the clinical efficacy of this system. We report an initial series of five cases utilizing the Resonance stent for extrinsic ureteral compression in advanced malignancy.

## MATERIALS AND METHODS

Upon Institutional Review Board approval, we conducted a retrospective review of all cases involving utilization, removal, or manipulation of a metallic full-length ureteral stent over a 20-month period by a single surgeon. Five case files were reviewed, and pre- and postoperative course for each case is reported. Patients were initially scheduled for follow-up at 3-month intervals after Resonance stent placement, but were uniformly seen more frequently to manage symptoms and complications.

## RESULTS

Table 1 details the course of the five patients undergoing Resonance ureteral stent placement in this series. All had metastatic abdominopelvic malignancies (three cervical, one anal squamous cell carcinoma, and one prostate + renal cell carcinoma). Three patients had bulky retroperitoneal malignancy on CT imaging. All patients presented with hydronephrosis and oliguria, pain, and/or renal failure. Standard 6 or 7 Fr ureteral stents (Percuflex®; Boston Scientific, Natick, MA), 10 Fr ureteral stents placed antegrade, and/or long-term ureteral stents (Silhouette®; Applied Medical, Rancho Santa Margarita, CA) had been placed and failed to provide long-term relief from obstruction. Percutaneous nephrostomy tubes (PCNT) were indwelling in two patients at the time of Resonance ureteral stent placement.

**TABLE 1**
**Preoperative Patient Characteristics**

| Patient # | Etiology of Obstruction | RP Lymphadenopathy | Previous Stents or Exchange | Prior Renal Surgery/Failure | Resonance Stents Placed | Intraoperative Complications with Resonance Placement |
|---|---|---|---|---|---|---|
| 1 | Metastatic prostatic/ renal malignancy | Yes | 3 | Left nephrectomy | 6F x 26 cm | None |
| 2 | Metastatic cervical malignancy | (Not reported) | 5 | No | Bilateral 6F x 24 cm | None |
| 3 | Metastatic cervical malignancy | Yes | 4 | No | Bilateral 6F x 24 cm | Initial overadvancement, ureteroscopic retrieval |
| 4 | Metastatic cervical malignancy | Yes | 2 | Left 25% renal function | Left 6F x 26 cm | Initial overadvancement, ureteroscopic retrieval |
| 5 | Metastatic squamous cell carcinoma | No (ovarian + vaginal metastasis | 2 | No | Bilateral 6F x 24 cm | None |

All five patients developed post–stent placement flank discomfort, two gross hematuria, three renal failure, and four renal obstruction. Four patients (80%) developed urinary tract infections (UTIs) within 4 months of stent placement. Resonance stent obstruction was demonstrated by various imaging modalities (demonstrating absence of antegrade urine or contrast flow into the bladder despite proper stent positioning), including antegrade pyelogram (Figs. 1 and 2), diuretic nuclear renography, and CT urogram. No obstruction was due to apparent extrinsic ureteral compression after stent placement.

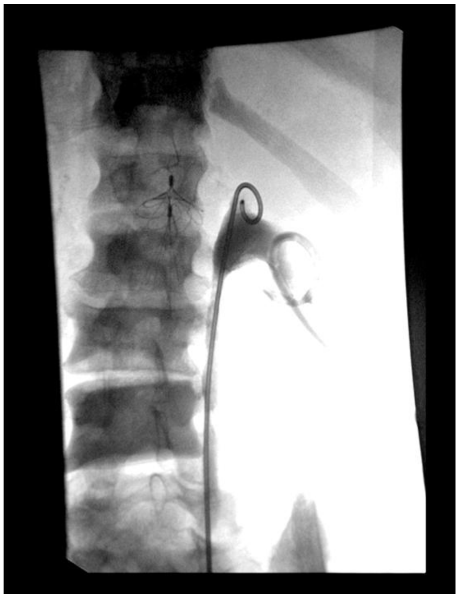

**FIGURE 1.** Case 1. Stent obstruction at 63 days; antegrade nephrostogram with absence of antegrade contrast flow.

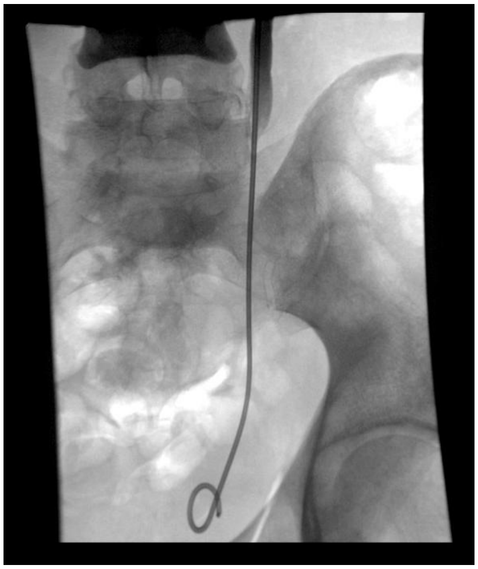

**FIGURE 2.** Case 2. Left hydroureteronephrosis with little antegrade flow, 2.5 months post–ureteral stent placement.

Repositioning of the Resonance stent was necessary at the time of placement in two patients. One of these patients also developed postoperative Resonance stent migration, with the distal coil protruding from the urethral meatus at the time of presentation to her medical oncologist. She required right Resonance stent removal and replacement under anesthesia (with confirmation of the normal position of the left stent). This was again complicated by overinsertion of the ureteral stent into the distal intramural ureter, requiring ureteroscopic retrieval and repositioning. She redeveloped obstruction and stent pain, and had both her Resonance stents removed. Two other patients also had Resonance stents removed.

Four (80%) patients required PCNT placement post–Resonance stent insertion, and three (60%) required Resonance stent removal and an alternative means of urinary tract drainage within 4 months of placement due to obstruction, intractable pain, or migration. Four patients (80%) died of their primary malignancy <12 months after metallic stent placement.

## DISCUSSION

Malignant extrinsic ureteral compression and obstruction truly poses a challenge to medical and surgical teams. Further, it bears a grim prognosis with median survival of only 3–7 months[4]. Complex medical issues are inherent in these patients undergoing chemotherapy, and particular attention to preservation of renal function and avoidance of infectious complications is necessary. Additionally, awareness of treatment side effects and impact on quality of life is paramount.

Standard polymer double-J, self-expandable metal, balloon-expandable, and thermo-expandable stents have all demonstrated limited effectiveness in treating malignant extrinsic ureteral obstruction (Table 2)[5,6]. Recently, metallic full-length coiled ureteral stents have demonstrated improved long-term patency rates in patients without significantly bulky pelvic disease. Wah et al. reported long-term urinary drainage (up to 12 months) in 14 of 17 (82%) stents placed in patients with malignant obstruction[7]. Liatsikos et al. reported 100% patency (mean follow-up 8.5 months) in 25 patients with malignant extrinsic compression[8]. While our sample size is small (five patients), we believe our much inferior results warrant presentation in view of the fact that other series have also reported less definitive results[9]. Nagele et al. reported stent removal in seven of 13 (54%) collecting systems with malignant obstruction[9]. Reasons for removal included persistent hematuria (one), severe dysuria (two), encrustation with obstruction (two), and obstruction with no apparent etiology (two). Our series observed similar complications.

**TABLE 2**
**Reported Results of Stents in Treatment of Malignant Extrinsic Ureteral Obstruction**

| Publication | Stents (n) | Mean Follow-Up | Tolerated | Etiology of Failure |
|---|---|---|---|---|
| Wah et al., 2007[7] | 17 | 1 week to 12 months* | 14 (82%) | Bulky pelvic disease (3) |
| Nagele et al., 2008[9] | 13 | 8.6 months | 6 (46%) | Persistent hematuria (1), severe dysuria (2), encrustation (2), idiopathic obstruction (2) |
| Liatsikos et al., 2009[8] | 25 | 8.5 months | 25 (100%) | None |
| Current series | 5 | 4.6 months | 1 (20%) | Obstruction (4) |

* Individual duration of follow-up not provided in referenced study.

It is well documented that indwelling ureteral stents may cause significant urinary symptoms and a reduction in quality of life[10]. Patients with ureteral stents may have more irritative urinary symptoms and local discomfort than patients with PCNT drainage[11]. While there are no published studies demonstrating a material or construction technique that provides a painless stent, thinner, more flexible stents have been found to be more comfortable overall[12]. Previous Resonance stent studies have suggested that improper stent length may correlate with increased patient discomfort. In one such study, Resonance stents 26 cm in length were associated with increased patient discomfort in patients <170 cm in height[9]. In the current study, stent length was determined by direct measurement of the ureter intraoperatively or by attending surgeon discretion. Retrospectively, all patients <170 cm in height received stents 24 cm in length, while those >170 cm received 26-cm stents. Despite this, improper stent length may have contributed to patient discomfort as direct measurement of straight ureter length on preoperative imaging has been shown to be a more accurate predictor of appropriate stent length than height[13].

Of peculiar interest is the observation of stent obstruction shortly after stent placement in 80% of patients described (Table 3). Radiologic and/or nuclear medicine studies, as well as clinical laboratory testing, confirmed obstruction and PCNT placement alleviated obstruction in all cases. No case had radiographic evidence of obstruction due to extrinsic stent compression. Prior *in vivo* testing of the Resonance ureteral stent has demonstrated decreased flow characteristics in an animal model[14]. Other studies have shown that Resonance stents placed in patients with large pelvic tumor burdens may become nonfunctional secondary to elevated intravesical pressure[7]. This increased resistance to flow, coupled with other factors, may have produced the observed obstructions.

**TABLE 3**
**Postoperative Course**

| Patient # | Stent Migration | Post-Resonance UTI | Postoperative Obstruction Prior to 12 months | Time Left Indwelling | Reason for Removal |
|---|---|---|---|---|---|
| 1 | No | Yes: 1, 2 months | 2 months | 2 months | Pain, gross hematuria, obstruction |
| 2 | No | Yes: 1, 2 months | 2.5 months | 7 months | Not removed, patient expired |
| 3 | Yes | Yes: 4, 5, 6 months | 4 months | 6 months total | Pain, obstruction |
| 4 | No | No | No (expired) | 4 months | Not removed, patient expired |
| 5 | No | Yes: 1, 2, 4 months | 4 months | 4 months | Pain, obstruction |

Prior investigators have also concluded that further studies are warranted to identify independent predictors of ureteral patency after the application of a metal stent for ureteral obstruction[5,6]. Given that all four patients suffering from Resonance stent obstruction had culture-proven UTIs in the months following placement, UTI may be a factor contributing to or causing metal stent obstruction. There are plausible reasons this may be the case. Studies assessing bacterial adherence to prosthetics used in the genitourinary system have indicated that up to 100% of patients using long-term ureteral stents for malignant extrinsic compression may have permanent bacterial stent colonization[15]. Novel strategies are in development to reduce the incidence of this phenomenon[16,17,18]. UTIs are associated with an increased turnover in epithelial cells and a subsequent increase in debris formation in the urinary tract. Cellular debris production, accumulation, and biofilm formation have been proposed as mechanisms for stent obstruction and encrustation[3,18,19,20]. A previous study comparing Resonance stent utility in patients with both malignant extrinsic compression and benign disease reported encrustation rates as high as 22% after only 8.5 months[8]. Other studies have shown encrustation of Resonance stents as early as 2 months[9]. The amount of biofilm deposition is proportional to the available inert surface area for binding. Biofilm formation and obstruction of stents used in the biliary system are persistent problems due to the microbe-rich microenvironment in the duodenum, and novel lumenless biliary stents with decreased surface area have been developed to decrease rates of obstruction associated with biofilm formation[21]. Similar ureteral stent development and design is an area of active investigation.

Regarding the surface area of ureteral stents, a standard 6F × 24 cm double-J ureteral stent with a wall thickness of 0.5 mm would have a total surface area of approximately 2262 $mm^2$ exposed to the urinary tract, with 1508 $mm^2$ attributable to the exterior of the cylindrical tube and 754 $mm^2$ to the interior. A similar 6F × 24 cm Resonance ureteral stent, manufactured of tightly coiled 0.2-mm diameter alloy wire would require approximately 1,200 individual turned coils of wire over a 24-cm length. A total length of wire of approximately 7.5 m would be utilized in this example, resulting in an exposed total surface area of wire of 4737 $mm^2$. No encrustation was present on Resonance stents in the current series. However, the over twofold increase in exposed surface area for adherence of biofilm and debris induced by infection, potential progression to encrustation, and decreased flow rates through the Resonance system (documented in experimental models) might be contributing factors for stent obstruction observed in this series. Recurrent Resonance stent obstruction has also been reported as a complication of intraureteral tumor extension, with obstruction occurring as quickly as 12 days postoperatively[22].

Patients with benign ureteral obstruction may be at increased risk of acute postintervention obstruction. One study reported that eight of 18 patients (44%) required intervention 2–12 days after stent placement, while stents remained patent in 100% of patients with malignant obstruction[8].

Resonance stents, as well as metal-reinforced stents from other manufacturers, are more resistant to extrinsic compression than nonmetal reinforced ureteral stents[23,24]. No patient in our series demonstrated evidence of stent obstruction due to extrinsic ureteral compression. Several patients, however, had previously obstructed polymer construction stents via ureteral compression based on imaging studies. Thus, the utility of all-metal or metal-reinforced ureteral stents for preventing compressive complications in advanced retroperitoneal or pelvic malignancy is clear. However, in our series these stents were not without the possibility of obstruction with infection.

Several series have reported ureteral stent migration incidence ranging from 1 to 4.2% of patients[25,26,27,28]. Migration was observed in one patient (20%) in our series, and required reoperation for replacement and repositioning. It is not clear what, if any, specific risk factors were associated with stent migration in this patient. To date, no other reports of Resonance stent migration have been published. Other reports have been published regarding migration rates and performance of other coated metallic ureteral stents; however, these reports are of stents of different design and construction and are not easily generalizable to this series[29,30].

In this case series, 80% of patients required PCNT placement after stent failure. PCNTs may be placed rapidly and provide definitive kidney drainage. However, only patients with cancers most likely to respond to ongoing palliative therapy (some colon), or are slowly progressing (prostate), may benefit from the procedure. In patients adverse to PCNT drainage, a safe and effective alternative is placement of two ureteral stents in a single ureter[31].

## CONCLUSION

The Resonance ureteral stent is clearly resistant to extrinsic ureteral compression, as documented in the literature. Its relative ease of insertion and longer indicated indwelling time are attractive potential attributes, especially in patients with extrinsic ureteral compression. While initial reports of use of the Resonance stent are promising, follow-up was limited. In this small series, four of five patients (80%) had clinical and/or radiologic evidence of ureteral obstruction despite Resonance stent placement, requiring removal of the device shortly after insertion. Patients receiving Resonance stents should undergo frequent monitoring as there appears to be a significantly increased risk of obstruction in patients with UTIs after placement. Additional, larger, multi-institutional studies are warranted in order to demonstrate the overall clinical utility of the Resonance stent placement in this relatively small patient population.
